# Depressive-like behavioral profiles in captive-bred single- and socially-housed rhesus and cynomolgus macaques: a species comparison

**DOI:** 10.3389/fnbeh.2014.00047

**Published:** 2014-02-19

**Authors:** Sandrine M. J. Camus, Céline Rochais, Catherine Blois-Heulin, Qin Li, Martine Hausberger, Erwan Bezard

**Affiliations:** ^1^Institut des Maladies Neurodégénératives, Université de Bordeaux, UMR 5293Bordeaux, France; ^2^Ethologie Animale et Humaine, Université de Rennes 1, CNRS, UMR 6552Rennes, France; ^3^Motac Neuroscience Ltd.Manchester, UK; ^4^Institute of Lab Animal Sciences, China Academy of Medical SciencesBeijing, China

**Keywords:** major depressive disorder, animal model, non-human primate, behavior, ethological methodology

## Abstract

**Background:** To unravel the causes of major depressive disorder (MDD), the third leading cause of disease burden around the world, ethological animal models have recently been proposed. Our previous studies highlighted a depressive-like profile among single- and socially-housed farm-bred cynomolgus macaques. Although phylogenetically close, cynomolgus and rhesus macaques, the two most commonly used macaque species in biomedical research, differ on several levels such as patterns of aggression, reconciliation, temperament, or dominance styles. The question of whether one captive macaque species was more vulnerable than another in the development of a pathological profile reminiscent of MDD symptoms was explored.

**Methods:** Behavioral data (including body postures, orientations, gaze directions, inter-individual distances, and locations in the cage) were collected in farming conditions. Using an unbiased validated ethological scan-sampling method, followed by multiple correspondence and hierarchical clustering analyses, 40 single- and 35 socially-housed rhesus macaques were assessed. Independently, for each housing condition, inter-species comparisons were made with previously acquired data on farm-bred cynomolgus monkeys.

**Results:** Consistent with our previous studies, we found depressive-like characteristics (e.g., inactivity, low level of investigation and maintenance, long time spent inactive while facing the wall) among single- and socially-housed rhesus macaques. Species-specificities were reported in non-depressive time budgets and in the prevalence of the pathological profiles.

**Conclusions:** Our results suggest that rhesus may be more vulnerable to developing a despair-like state than cynomolgus macaques, both in single- and in social-housing conditions. Therefore, rhesus macaques are more suitable for use as a “spontaneous” model of depressive disorders.

## Introduction

The underlying factors causing major depressive disorder (MDD) remain poorly understood, while the heterogeneity of depressive symptoms (APA, 1994) and the lack of acknowledged biomarkers prevent researchers from validating the numerous existing animal models (Nestler and Hyman, 2010; Berton et al., 2012). Several approaches have been described, namely induced, stress-associated or ethological models. The induced models have mostly been developed in rodents but, although sensitive to classic antidepressant treatment, these models lack construct validity (e.g., O'Neil and Moore, 2003; Matthews et al., 2005; Haenisch and Bonisch, 2011). Stress-associated models mimic the conditions theoretically associated with the onset on MDD (de Kloet et al., 2005; Daskalakis et al., 2012; Nederhof and Schmidt, 2012). However, their interesting construct value is devalued by the large variability across settings (e.g., chronic mild stress Willner, 1997). Inspired by ethological findings, “naturalistic” settings (i.e., animals are observed in their usual, yet captive, environment without any testing paradigms) are also investigated. Macaque monkeys have been studied in response to ecologically valid challenges (e.g., hierarchical ranking reorganizations Shively and Willard, 2012 or impoverished maternal care due to unpredictable food availability Kalin and Shelton, 2003). These species showed depressive- or anxiety-like behaviors, such as increased levels of immobility, a slumped body posture in which they appear to be withdrawn from the environment. Juvenile monkeys also expressed decreased play and investigation behaviors.

Availing of the animal “spontaneous” modifications of their behavioral repertoires (in opposition to “induced” changes requiring invasive methods and direct manipulations of the individuals) and using ethological observation methods, we have previously highlighted atypical behavioral profiles reminiscent of depressive-like symptoms (APA, 1994) among single- and socially-housed cynomolgus macaques living in Chinese breeding facilities (Camus et al., 2013a,b). Although obviously phylogenetically close, cynomolgus and rhesus macaques present species-specific characteristics, regarding patterns of aggression, reconciliation, dominance, or temperament (Clarke and Mason, 1988; Thierry et al., 2000; Sussman et al., 2013). For instance, rhesus macaques are highly hierarchical and nepotistic, while cynomolgus exhibit more affiliative behaviors and their aggressive encounters are less intense (Thierry et al., 2000). Following maternal separations, both rhesus and cynomolgus infants showed increased levels of locomotion, vocalization and defecation, followed by decreased play, locomotion and a despair-like state (Seay and Gottfried, 1975). However after reunion, the elevated rate of contacts with their mother persisted longer in cynomolgus monkeys compared to rhesus, suggesting greater negative impact of early maternal separation in the cynomolgus (Seay and Gottfried, 1975). Other differences have been reported, such as rhesus being aggressive toward humans while cynomolgus macaques are more cautious and fearful (Clarke and Mason, 1988; Sussman et al., 2013). Social functioning differing between these species and being an important impaired feature in MDD, it would not be surprising that rhesus and cynomolgus macaques cope differently with suboptimal captive environments by expressing distinct modifications of their behavioral repertoires.

With the exception of research on temperament and personality, no recent direct comparative studies have investigated whether one macaque species is more susceptible to develop a pathological profile reminiscent of MDD symptoms than others in a captive environment. Hence our aim was two-fold: (i) identify atypical behavioral profiles, possibly reminiscent of MDD, displayed by rhesus macaques in single- and social-housing conditions using an unbiased ethological analysis and (ii) investigate inter-species (rhesus vs. cynomolgus) differences regarding time budgets and depressive-like symptomatology in such farm-bred animals. The results are presented in three distinct studies, describing global and individual profiles of (i) single- and (ii) socially-housed rhesus macaques, respectively, and (iii) comparing cynomolgus and rhesus monkeys in both housing situations (i.e., single-housed rhesus vs. cynomolgus monkeys and socially-housed rhesus vs. cynomolgus monkeys). The 3rd study was performed by comparing newly acquired data in a rhesus group with our previously published data on cynomolgus monkeys (Camus et al., 2013a,b).

## Materials and methods

### Ethics statement

The institutional animal care and use committee of the Institute of Lab Animal Science of Chinese Academy of Medical Science approved this study. The housing conditions were in compliance with the guidelines of the Beijing Forestry Office (People's Republic of China) and correspond to standard practices in operation in breeding facilities providing macaques to the whole Japanese, American and European toxicology industry and research laboratories. Our study availed of such conditions. We did not require longer single-housing period or any environmental changes compared to what is commonly done in those facilities. Veterinarians skilled in the healthcare and maintenance of non-human primates supervised animal care. No animal was harmed or killed in the course of the experiments.

### Animals and housing conditions

Forty male and thirty-five female rhesus monkeys (*Macaca mulatta*) and forty male and eighty female cynomolgus monkeys (*Macaca fascicularis*) living in three distinct Chinese breeding farms were included in this study. Data from the cynomolgus populations have been previously published in Camus et al. (2013a,b) whereas data from the rhesus monkeys are newly presented here. The animal information and housing conditions are described in Table 1. Toys and fruits were provided as environmental enrichment, as well as swings in the social groups. A schematic plan of a common social-housing cage is available in our previous paper (see Figure S1 in Camus et al., 2013b).

**Table 1:** **Rhesus and cynomolgus single- and social- housing conditions and animal information**.

	**Rhesus monkeys**		**Cynomolgus monkeys**	
Breeding farm	Institute of Beijing Xierxin Biology Resource	Institute of Beijing Xierxin Biology Resource	Hannan Jingang Laboratory Animal Corporation	Fangcheng Gang Spring Biological Technology Development Corporation
Location	Beijing	Beijing	Hannan Province	Guangxi province
Observed sample size	40 ♂	35 ♀	40 ♂	80 ♀
Mean age ± s.e.m (years old)	4.4 ± 0.1	9.6 ± 0.6	3.5 ± 0.01	5.9 ± 0.1
Pre-weaning	1 ♂/multi♀ group	1 ♂/multi♀ group	1 ♂/multi♀ group	1 ♂/multi♀ group
Weaning	6 months old	6 months old	6 months old	6 months old
Peer-housing	Until 3 years of age	Until 3 years of age	Until 3 years of age	Until 3 years of age
Post peer-rearing housing conditions	Indoor single cage	Indoor/outdoor social group	Indoor single cage	Indoor social group
Cage dimensions	L70 × W55 × H75 cm	Outdoor: L5.1 × W2.8 × H2.9 m	L70 × W60 × H80 cm	Indoor: L3.50 × W7 × H3 m
Cage number	10 cages per room 4 observed rooms	8 observed cages: 1 ♂/8–9 ♀ per cage	10 cages per room 4 observed rooms	8 observed cages: 1 ♂/17–27 ♀ per cage
Stability pre-observation	9 months	12 months	9 months	9 months
Social stimuli	V A O m	V A O M	V A O m	V A O M
Feeding schedule	SAUE Ltd Old World Monkey pellets twice and fruit once daily	SAUE Ltd Old World Monkey pellets twice and fruit once daily	SAUE Ltd Old World Monkey pellets 3 times and fruit once daily	SAUE Ltd Old World Monkey pellets twice and fruit once daily
Water	*Ad libitum*	*Ad libitum*	In a water tray filled at each feeding times	*Ad libitum*
Natural lighting	Through windows	Through wire-mesh roof	Through windows	Through wire-mesh roof

*V, Visual; A, auditory; O, olfactory; manual (m, through the top of the cage or M, direct contact); ♂, male; ♀, female*.

### Behavioral assessment

Following a 4-h habituation phase performed one day before the beginning of the observations, macaque behavior was video-recorded and then scored (single-housing), or scored and recorded live (social-housing), by two trained observer (SC and CR; inter-observer reliability: Spearman rank order correlation *R* = 0.86) outside the feeding and cleaning times, as previously described (Camus et al., 2013a,b).

#### Single-housing (Camus et al., 2013a)

For each room, data were collected in a randomized order at three time points (morning, noon, and afternoon), on 2 non-consecutive days (6 sessions per individual). The observer was facing the door or window at all times rather than the cages and looked at the camera screen rather than at the individuals (a gaze directed to a macaque's eyes being interpreted as a threat).

#### Social-housing (Camus et al., 2013b)

For each group, data were collected in a randomized order at two time points (morning and afternoon) on 6 consecutive days. More sessions were needed, as such housing allowed more complex behaviors. The observer was sitting 1 m away from the front of the outdoor cages.

In both conditions, we used a scan-sampling method, appropriate for time budgeting (Altmann, 1974), in which behavioral parameters were assessed every 2 min during 30-min sessions resulting in 90 scans per male, or every 6 min during 2-h sessions resulting in 240 scans per female. We focused on behavioral profiles rather than single items and used two repertoires: one reporting the interaction with the environment (see Table S1) and one describing the position within the environment (see Table S2) according to published protocols (Camus et al., 2013a,b).

### Factor analyses

Following our previously described procedures (Camus et al., 2013a,b), data from single- and socially-housed individuals were separately submitted to multiple correspondence analysis (MCA; SPAD^©^ 7.4, Coheris) that uses chi-square criterion to assess differences and similarities between frequencies of qualitative variables (Montaudouin and Le Pape, 2004). We used grouped behaviors, grouped body postures, body orientations, and locations as active variables. A hierarchical clustering analysis was then performed on the individuals' coordinates to describe inter-individual similarities (Henry et al., 2005). For each resulting cluster of individuals, the mean occurrence percentage of each behavioral item was calculated and reported on radar graphs. The “behavioral diversity” was assessed as the mean number of distinct behaviors observed during the scans. The “behavioral switch” between successive scans was calculated for single-housed individuals, using a score for each scan: 0 if the behavior was the same as in the previous scan, or 1 if it was different; the scores were added up within one session and transformed in a percentage with regard to the 15 scans of a session. Depressive-like profiles were identified according to similarities with the previously described depressive-like profiles among cynomolgus monkeys (Camus et al., 2013a,b) resulting from comparisons with the MDD criteria described in the Diagnostic and Statistical Manual of Mental Disorders (DSM-IV; APA, 1994) (see Table 2).

**Table 2:** **Summary of depressive-like features reported in single- and socially-housed rhesus and cynomolgus macaques and similarities with human depressive symptoms**.

**Human major depressive disorder DSM-IV criteria (APA, 1994) and verbal reports**	**Macaque depressive-like profile Behavioral daily life home cage observations**
1. Depressed mood	/
2. ↘ interest in most activities or ↘ pleasure in most activities	↘ Investigation, maintenance, social behaviors, behavioral diversity
	↗ Gaze and body oriented toward the wall, location in the back (perspective: sucrose consumption)
3. ↘ or ↗ weight/appetite	↘ Feeding
4. Insomnia or hypersomnia	↗ Inactivity
5. Psychomotor agitation or retardation	↘ Locomotion, poorer posture, and location diversity
6. Fatigue or loss of energy	↗ Inactive while slumped
7. ↘ Ability to concentrate or indecisiveness	(perspective: CANTAB)
8. Feelings of worthlessness/inappropriate guilt	/
9. Recurrent thoughts of death	/

*Abbreviations: ↘, decreased; ↗, increased; and CANTAB, Cambridge Neuropsychological Test Automated Battery*.

### Statistical analyses

The statistical analyses were conducted using Statistica© 8.0 (StatSoft, Inc.). For each population (rhesus, cynomolgus, single- or socially-housed) data collected were not normally distributed. Therefore, non-parametric Mann-Whitney *U*-tests were used to compare behavioral variables between two populations. A correction for small group size was applied when the group contained less than 10 individuals.

Spearman rank correlations were performed to assess correlations between age, weight and the collected variables within groups of individuals. As numerous tests were performed, a Bonferroni adjustment was applied to keep the type I error constant. The accepted P level becomes the α probability divided by the number of hypothesis tests: 0.00040 (124 hypotheses) and 0.00028 (177 hypotheses) in single- and social-housing conditions, respectively.

## Results

### Study 1: global and cluster time budgets in single-housed rhesus monkeys

The main behaviors expressed by the 40 **single-housed rhesus monkeys** were inactivity, maintenance behaviors and investigation (Table 3, “Rhesus total” column). They were mostly seated and displayed a slumped posture in 14.13% (±2.07%) of the scans. When slumped, the monkeys expressed maintenance behaviors or were inactive. They faced the wall in 56.47% (±4.21%) of the scans and were mainly inactive when doing so. They were mainly at the back bottom side of the cages and directed their gaze at the still and living environment.

**Table 3:**
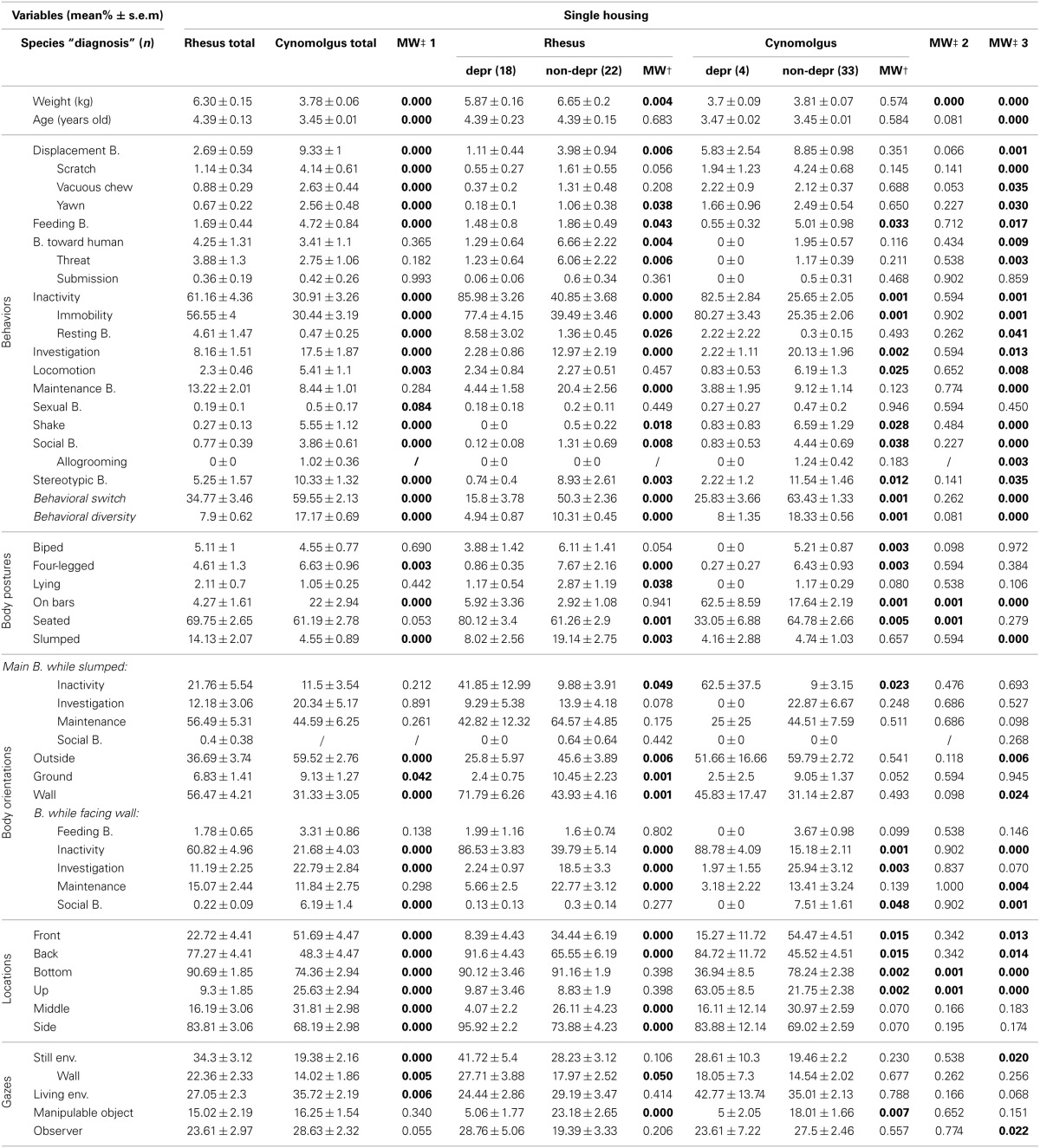
**Single-housed rhesus and cynomolgus monkey total, depressive-like and non-depressive time budgets**.

*depr, Depressive-like; non-depr, non-depressive; B, behavior; env, environment; MW, Mann–Whitney U-test p-values; †, depr vs. non-depr; ‡, rhesus vs. cyno; 1, among total population; 2, among depressive-like animals, 3, among non-depr animals. U statistics are reported in Table S3. Bold values are statistically significant (p < 0.05)*.

**Figure 1:**
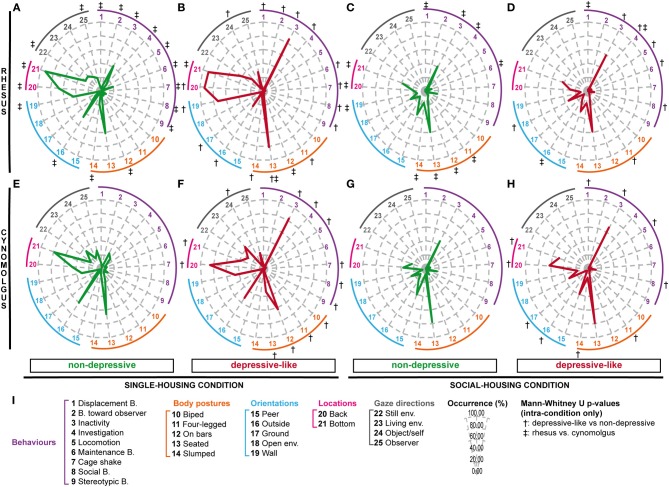
**Depressive-like and non-depressive behavioral profiles resulting from hierarchical cluster analyses among single- and socially-housed rhesus and cynomolgus macaques**. Rhesus **(A–D)** and cynomolgus **(E–H)** monkeys were observed in single- **(A,B,E,F)** or social-housing **(C,D,G,H)** conditions. Following multiple component and hierarchical cluster analyses, the observed populations were divided into depressive-like **(B,D,F,H)** and non-depressive **(A,C,E,G)** groups, containing *n*_A_ = 22, *n*_B_ = 18, *n*_C_ = 29, *n*_D_ = 6, *n*_E_ = 33, *n*_F_ = 4, *n*_G_ = 75, and *n*_H_ = 5 animals. The mean percentages of occurrence were calculated among the 8 groups for a selection of collected variables **(I)**. Each axis of the radar indicates the mean percentage of occurrence for a given variable: a behavior (from 1 to 9), a body posture (from 10 to 14), a body orientation (from 15 to 19), a location in the cage (20 and 21) or a gaze direction (22–25). The abbreviations “B.” and “env.” stand for “behavior” and “environment.” Significant intra-condition *p*-values (<0.05) after Mann-Whitney *U*-tests are indicated by crosses: † for comparisons of depressive-like vs. non-depressive animals (**A** vs. **B**, **C** vs. **D**, **E** vs. **F**, or **G** vs. **H**) and ‡ for comparisons of rhesus vs. cynomolgus animals (**A** vs. **E**, **B** vs. **F**, **C** vs. **G**, or **D** vs. **H**). See Tables 3 (single-housed monkeys) and 4 (socially-housed monkeys) for detailed time budgets and Mann-Whitney *U*-test *p*-values.

In accordance with our previous findings among single-housed cynomolgus monkeys (Camus et al., 2013a), rhesus individual values for several collected parameters presented a wide distribution (e.g., Figure S1A). We apprehended such great inter-individual variability using multiple correspondence analysis (MCA) and hierarchical clustering (see Figure S2), which allowed us to identify nine clusters of individuals displaying similar profiles. Regarding our aim and the depressive-like profiles reported in our previous cynomolgus studies (Camus et al., 2013a,b), three clusters (*n* = 18) were of a particular interest. The six other clusters (*n* = 22) differed on several parameters but had in common the fact that they did not display depressive-like features. We pooled these three depressive-like and the six non-depressive clusters, respectively. Salient results are displayed on radar graphs in Figures 1A,B while comprehensive statistical analyses and detailed time budgets are presented in Table 3 (“Rhesus depr” and “Rhesus non-depr” columns).

Depressive-like rhesus monkeys weighted significantly less than the non-depressive ones. They expressed a higher level of inactivity (both immobility and rest), and significantly lower levels of displacement, feeding, threat, investigation, maintenance, social, and stereotypic behaviors (SB), in addition to lower mean behavioral switch and behavioral diversity. Regarding body postures, depressive-like monkeys were seated more often than their peers but displayed less often the slumped posture. However, when slumped, depressive-like monkeys were inactive significantly more often than the non-depressive ones. Their body was oriented toward the wall more often and this orientation was associated with inactivity significantly more often in the depressive-like rhesus monkeys. The depressive-like monkeys were located more often on the back side of the cages compared to the non-depressive ones. Finally, the gazes of depressive-like monkeys were directed more frequently toward the walls and less frequently toward manipulable objects than their non-depressive counterparts (see Figure S1A, for individual values).

### Study 2: global and cluster time budgets in socially-housed rhesus monkeys

The main behaviors expressed by the 35 **socially-housed rhesus monkeys** were inactivity, social, and maintenance behaviors (Table 4, “Rhesus total” column). They were mostly seated and displayed a slumped posture in 17.10% (±1.68%) of the scans. When slumped, the monkeys expressed maintenance, inactivity, or social behaviors. They faced mainly peers or the open environment. They were mostly located on sitting benches on the side of the cages. They spent 35.33% (±1.97%) of the time at arm-length distance and 27.72% (±2.09%) between 1 and 3 m from their peers.

**Table 4:**
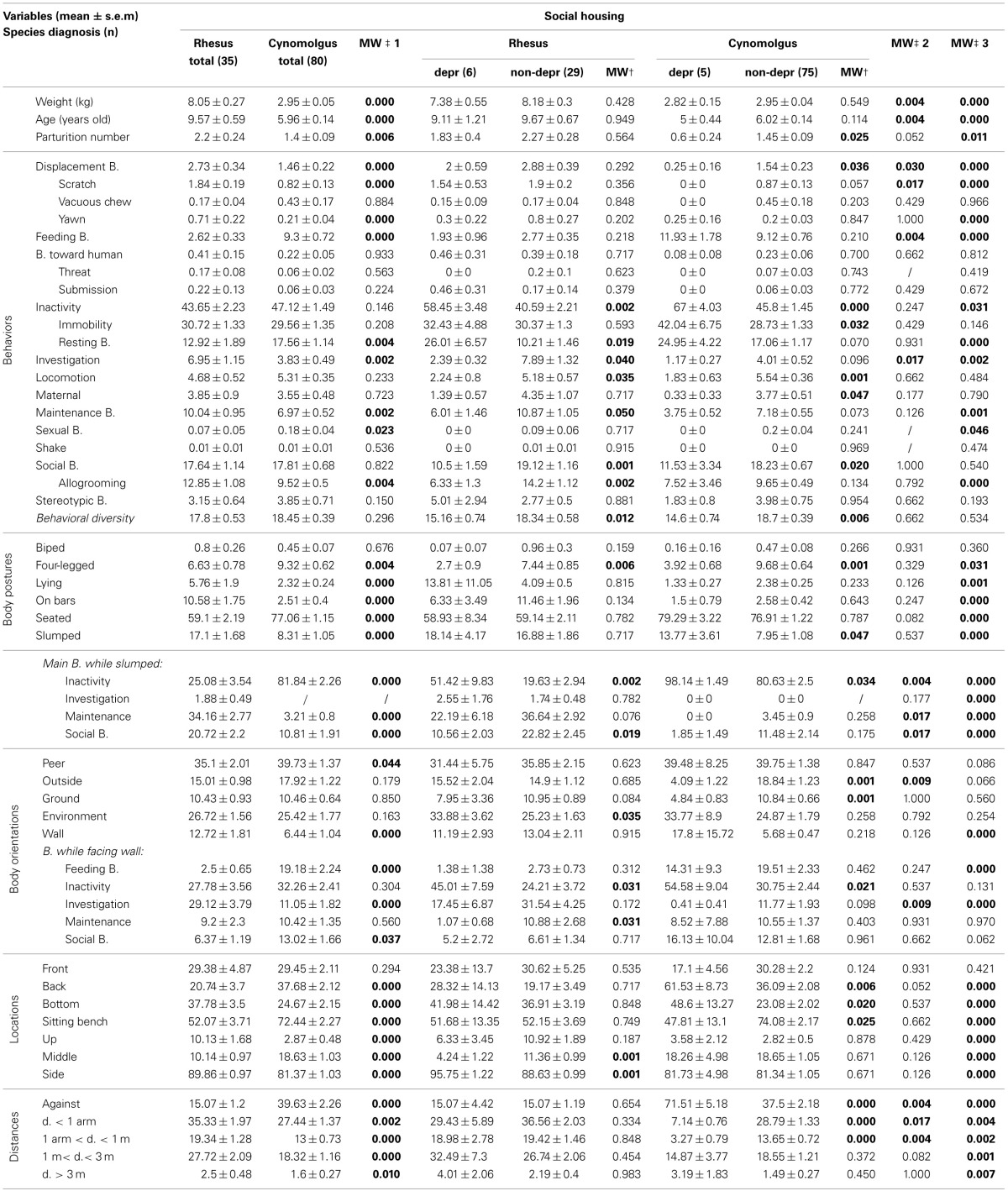
**Socially-housed rhesus and cynomolgus monkey total, depressive-like and non-depressive time budgets**.

*depr, Depressive-like; non-depr, non-depressive; B, behavior; env, environment; d, distance; MW, Mann-Whitney U-test p-values; †, depr vs. non-depr, ‡, rhesus vs. cyno; 1, among total population; 2, among depressive-like animals; 3, among non-depr animals. U statistics are reported in Table S4. Bold values are statistically significant (p < 0.05)*.

Similarly to the single-housed population, wide distributions were reported among several variables (e.g., Figure S1B). Animals were therefore submitted to MCA and hierarchical clustering as well, resulting in eight clusters (see Figure S3). Three clusters (*n* = 6), similar to the depressive-like socially-housed cynomolgus monkeys (Camus et al., 2013b) on several points, were pooled as the depressive-like group, while the five remaining clusters (*n* = 29) constituted the non-depressive group. Salient results are displayed on radar graphs in Figures 1C,D while comprehensive statistical analyses and detailed time budgets are presented in Table 4 (“Rhesus depr” and “Rhesus non-depr” columns).

The depressive-like animals were more often inactive (especially resting) and expressed lower levels of investigation, locomotion, maintenance, and social behaviors (especially allogrooming) compared to non-depressive monkeys. Their behavioral diversity was significantly lower as well. Only the four-legged posture significantly differed between depressive-like and non-depressive socially-housed rhesus. However, depressive-like animals were more often inactive and less often in social interactions, compared to non-depressive ones, when displaying the slumped body posture. Depressive-like monkeys faced the open environment more than their non-depressive counterparts. When facing the wall depressive-like animals spent half of their time inactive while non-depressive monkeys expressed investigation or maintenance behaviors. Regarding the locations, depressive-like animals spent more time located on the sides of the cage compared to non-depressive ones. No significant difference was reported relative to the distances to the nearest peer (see Figure S1B, for individual values).

### Study 3: a species comparison

Data collected with the exact same methodology were used to obtain time budgets of single- (Figures 1E,F, Table 3, “cynomolgus” columns) and socially-housed cynomolgus monkeys (Figures 1G,H, Table 4, “cynomolgus” columns), described in our previous papers (Camus et al., 2013a,b). We will therefore focus on species differences. Since the differences displayed in the total populations (Tables 3, 4, “MW‡1” columns) might be biased by the pooled depressive-like and non-depressive individuals, we reported them as informative results. The most pertinent and reliable results were the inter-species differences among depressive-like (Tables 3, 4, “MW‡2” columns) or non-depressive (Tables 3, 4 “MW‡3” columns) animals separately, in both housing conditions (see Tables S3, S4 for Mann-Whitney *U*-test statistics in single- and socially-housed monkeys, respectively).

#### Time budgets differed between species when single-housed

Few inter-species differences were reported in the **depressive-like single-housed animals** (Table 3, “MW‡2” column). Rhesus were significantly heavier than the cynomolgus monkeys. No inter-species differences in behavior, body orientation, or gaze direction were reported. Depressive-like rhesus seated (thereby at the bottom of the cage) more often than cynomolgus monkeys which were mainly hanging on bars (thereby located in the upper part of the cage).

Conversely, many inter-species differences were highlighted in the **non-depressive single-housed population** in each category of collected parameter (Table 3, “MW‡3” column). Weight and age were significantly higher in rhesus monkeys. All behaviors, except submission to the observer and sexual behaviors, differed between rhesus and cynomolgus macaques. Non-depressive cynomolgus displayed more “on bars” posture, while rhesus were more often slumped. As a result of the posture, cynomolgus were more often located in the upper part of the cage whereas rhesus mostly stayed at the bottom of the cage. In addition, the percentage of time spent at the back of the cage was higher in rhesus compared to cynomolgus macaques. Rhesus spent more time facing the wall relative to cynomolgus which faced the exterior more often. Finally, non-depressive rhesus directed their gazes at the still environment more than cynomolgus monkeys which looked at the observer significantly more.

Since weight and/or age differed between the species in the single-housed monkeys, we tested whether these variables could impact the occurrences of other collected parameters. No significant Spearman rank correlations were reported in the depressive-like or non-depressive populations (data not shown).

#### Time budgets differed between species when socially-housed

Few inter-species differences were reported in the **depressive-like socially-housed animals** (Table 4, “MW‡2” column). Rhesus monkeys were significantly heavier and older than the cynomolgus monkeys. Depressive-like rhesus expressed more displacement, especially scratching, and investigation behaviors than cynomolgus monkeys that fed more often. No significant inter-species difference was reported relative to body posture. When slumped, however, rhesus expressed more maintenance and social behaviors compared to cynomolgus depressive-like monkeys that were more often inactive. Regarding body orientations, rhesus faced the exterior more often than cynomolgus macaques. Although the orientation toward the wall was similar in both species, rhesus investigated the cage more often than cynomolgus monkeys when facing a wall. The locations did not differ according to species. Finally, depressive-like rhesus monkeys stood within 1 m of their peers while cynomolgus monkeys spent more time against a congener.

As in the single-housing conditions, many inter-species differences were highlighted in the **socially-housed non-depressive population** (Table 4, “MW‡3” column). Weight, age, and parturition number were significantly higher in the rhesus monkeys. Several behaviors, including the 3 that differed in the depressive-like group, differed between rhesus and cynomolgus macaques. All postures, but the bipedal one, differed between the species. Rhesus were more often lying, on bars and slumped compared to cynomolgus monkeys. As in the depressive-like group, rhesus were more active than cynomolgus macaques when slumped. Non-depressive rhesus faced the wall more often than their cynomolgus peers but both species did not express the same behavior while doing so (i.e., rhesus investigated the cage whereas cynomolgus fed). Every location differed relative to species. Significant characteristics included being located at mid-depth, up, or down on the sides of the cage or on sitting benches at the middle back of the cage for rhesus and cynomolgus monkeys, respectively. Finally, while non-depressive cynomolgus spent more time against a peer, rhesus monkeys showed higher levels of every other distance category.

We also investigated the correlations between weight, age, parturition number, and the collected variables, since they differed between the species in the socially-housed monkeys as well. No significant Spearman rank correlations were reported in the depressive-like rhesus and cynomolgus groups (Table 5, “depressive-like” columns). In the non-depressive rhesus group, age was positively correlated with the parturition number but neither correlated with other collected parameters (Table 5, “rhesus non-depressive” column). Conversely, several correlations were reported in the non-depressive cynomolgus group (Table 5, “cynomolgus non-depressive” column). Age correlated positively with weight, parturition number, inactivity, and location on the sitting benches whereas it was negatively correlated with investigation, SB, the “on bar” posture and the locations “front,” “bottom,” and “up.” The parturition number was positively correlated with maternal behaviors and location on sitting benches and negatively correlated with feeding, investigation, “on bar” posture, facing the wall (especially while expressing maintenance behaviors) and “bottom” and “up” locations.

**Table 5:** **Significant Spearman rank correlations between weight, age, parturition number, and other collected parameters in socially-housed depressive-like and non-depressive macaques**.

**Species diagnosis (n)**	**Rhesus**						**Cynomolgus**					
**Variables**	**Depressive-like (6)**			**Non-depressive (29)**			**Depressive-like (5)**			**Non-depressive (75)**		
	**Weight**	**Age**	**PN**	**Weight**	**Age**	**PN**	**Weight**	**Age**	**PN**	**Weight**	**Age**	**PN**
Weight (kg)	1.000	0.212	−0.185	1.000	0.445	0.613	1.000	0.648	0.444	1.000	**0.429**	0.132
Age (years old)	0.212	1.000	0.393	0.445	1.000	**0.849**	0.648	1.000	0.000	**0.429**	1.000	**0.473**
Parturition number (PN)	−0.185	0.393	1.000	0.613	**0.849**	1.000	0.444	0.000	1.000	0.132	**0.473**	1.000
Feeding B	−0.771	−0.576	0.092	−0.040	0.005	0.226	−0.205	−0.632	−0.288	−0.118	−0.376	−**0.505**
Inactivity	−0.314	−0.941	−0.246	−0.220	−0.033	−0.126	0.102	0.000	0.866	0.173	**0.454**	0.390
Investigation	−0.116	0.184	−0.015	0.133	0.035	0.076	−0.461	0.316	−0.288	−0.288	**−0.540**	**−0.535**
Maternal B	−0.441	−0.469	0.572	0.484	0.155	0.419	0.181	−0.559	0.408	0.097	0.223	**0.609**
Stereotypic B	0.371	−0.394	−0.925	−0.165	−0.151	−0.162	−0.666	0.000	−0.866	−0.096	**−0.458**	−0.225
“On bars” posture	−0.371	−0.091	−0.617	−0.373	−0.547	−0.578	0.359	0.316	−0.288	−0.169	**−0.427**	**−0.447**
Body facing wall	0.428	0.516	−0.092	0.282	0.171	0.207	−0.564	−0.316	0.288	−0.201	−0.370	**−0.460**
Maintenance facing wall	0.304	0.826	−0.018	−0.031	0.347	0.328	−0.057	−0.530	0.645	0.004	−0.230	**−0.433**
“Front” location	−0.116	−0.770	−0.563	−0.433	−0.368	−0.485	−0.359	−0.316	−0.866	−0.121	**−0.412**	−0.152
“Bottom” location	−0.085	−0.030	0.308	0.064	0.132	0.031	−0.153	0.316	0.288	−0.328	**−0.530**	**−0.411**
“Sitting bench” location	0.085	−0.333	−0.092	0.123	0.096	0.218	0.153	−0.316	−0.288	0.322	**0.566**	**0.467**
“Up” location	−0.260	0.000	−0.579	−0.334	−0.532	−0.563	0.289	−0.081	0.148	−0.178	**−0.428**	**−0.419**

*B stands for behavior. Bold Spearman rank correlations were significant following a Bonferroni correction (p < 0.00028)*.

## Discussion

In this study, depressive-like profiles among both single- and socially-housed rhesus monkeys were identified, via home cage observations and subsequent multiple component and hierarchical analyses. Inter-species differences were detected regarding time budgets in both housing conditions by comparing current rhesus monkey datasets with our previously acquired cynomolgus datasets (Camus et al., 2013a,b). We highlighted species-specificity among the non-depressive populations while rhesus and cynomolgus depressive-like individuals did not differ much.

### Behavioral profiles in rhesus monkeys (studies 1 and 2)

Using distinct multiple component and hierarchical analyses, 3 single- and 3 socially-housed rhesus clusters drew our attention. We looked for the clusters which differed the most from the usual 40–45% of inactivity reported by Crockett et al. among captive macaques (Crockett et al., 1995), since a modified locomotive activity seems to be a recurrent characteristic between most models of depressive symptoms (e.g., forced swim test, learned helplessness, early-life, or social stress paradigms, for reviews see O'Neil and Moore, 2003; Nestler and Hyman, 2010). These six profiles were qualitatively and quantitatively very similar to the ones reported in our previous study investigating **depressive-like** profiles among single- or socially- housed cynomolgus macaques (Camus et al., 2013a,b). In each housing condition, when pooled, these clusters of interest displayed similar characteristics that significantly differed from the rest of the populations. Indeed, in both single- and social-housing conditions, these animals expressed a high level of inactivity, especially resting, and low levels of investigation, maintenance, social behaviors, resulting in a low behavioral diversity. These features recall symptoms of MDD according to the DSM-IV (APA, 1994), namely the decrease of interest in usual activities, the psychomotor slowdown, and energy loss (see Table 2). They were also behaviorally consistent with the depressed monkeys in Harlow's or Shively's studies, displaying inactivity with open eyes, a lack of responsiveness to environmental events, decreased locomotion, and self-grooming rates (Harlow and Suomi, 1974; Suomi et al., 1975; Shively and Willard, 2012). However, unlike the depressed monkeys from the literature, our depressive-like animals were not the ones displaying the most slumped postures. This finding was consistent with our cynomolgus study (Camus et al., 2013a,b). We therefore investigated the behaviors while slumped and found that depressive-like monkeys were often inactive in that posture. Added to the high level of inactivity while facing the wall, these profiles were concordant with the lack of interest for environmental stimuli suggested in “depressed” horses (Fureix et al., 2012).

In the single-housing condition, the lack of interest for usual activities and environmental stimuli was even more pronounced with many other decreased behavioral occurrences, high levels of body facing the wall, gaze directed at the wall and a clear favored location at the back of the cage. Moreover, the depressive-like animals weighed less and also fed less than the non-depressive animals, recalling another criterion of MDD (i.e., loss of weight and/or of appetite). This more severe depressive-like state was not surprising considering the suboptimality that single-housing represents for a gregarious species (Roder and Timmermans, 2002; Gilbert and Baker, 2011).

In the social-housing condition, locomotion and allogrooming were significantly lower in depressive-like compared to non-depressive animals. These two behaviors, predominant in the time budget of wild rhesus populations (Post and Baulu, 1978; Fooden, 2000), also suggested a decreased interest for usual activities, mimicking one major symptom of MDD (APA, 1994). Moreover, depressive patients have been reported to display fewer smiles and to invite others to social interactions less often than the controls (Geerts and Brune, 2009).

Conversely, the **non-depressive** single- and socially-housed monkeys expressed many wild-like behaviors, with a predominance of inactivity, maintenance, social and investigation behaviors as in free-ranging or captive rhesus macaques (Post and Baulu, 1978; Fooden, 2000), suggesting their ability to adapt to/cope with captivity. The hierarchical discrimination of several non-depressive clusters among both housing conditions have been discussed in our previous studies (Camus et al., 2013a,b) with regard to temperament, social hierarchy, age, parturition numbers, or early life experience, that have all been suggested as impacting the expression of behaviors in the literature (de Waal and Luttrell, 1989; Veenema et al., 1997; Thierry et al., 2000; Freeman and Gosling, 2010; Claessens et al., 2011; Shively and Willard, 2012; Sussman et al., 2013). Investigating the origin of such diversity was not our goal and would require another full set of studies.

These promising results suggest that, as Humans, a small proportion of farm-bred rhesus macaques develop an atypical behavioral profile, reminiscent of several symptoms of MDD, when subjected to stressful, yet common to every breeding facility, processes (Table 2). More investigations are of course needed to further characterize our potentially depressive-like profile: the sucrose preference test or the responses to a reward are indeed often used to study the core symptom of MDD, i.e., anhedonia (O'Neil and Moore, 2003). Cognitive impairments, i.e., attention deficit and/or decision-making difficulty, or the memory losses reported by depressive patients (APA, 1994), can also be studied with attention, motivation or memory tests using a complete test battery adapted from a human neuropsychological assessment battery (CANTAB), thereby allowing cross species comparisons (Crofts et al., 1999).

### Rhesus/cynomolgus inter-species differences (study 3)

The collection of behavioral data with the same methodology among single- and socially-housed cynomolgus populations (described in Camus et al., 2013a,b), allowed us to assess differences between species in both depressive-like and non-depressive individuals.

#### Inter-species differences in non-depressive animals

Although displaying many wild-like behaviors (e.g., investigation, maintenance behaviors, support shaking, or social behaviors) in proportions consistent with captivity data (Post and Baulu, 1978; Crockett et al., 1995; Fooden, 2000), many features significantly differed between rhesus and cynomolgus **non-depressive** monkeys. We propose that most of these differences can be associated with the differential **temperamental and socio-ecological** characteristics of these species.

For instance, the lower level of displacement behaviors (i.e., displayed in anxiogenic situations Tinbergen, 1952; Schino et al., 1996; Troisi, 2002) and higher level of threat toward the observer in single-housed rhesus was in accordance with several comparative studies on temperament describing them as aggressive and hostile in the presence of a passive human observer compared to the “fearful and cautious” cynomolgus monkeys (Clarke and Mason, 1988; Sussman et al., 2013). Single-housed rhesus monkeys were twice as inactive as cynomolgus macaques, leading to smaller behavioral switch rate and diversity. Interestingly, rhesus macaques could have compensated their lack of investigation and social behaviors by increasing the expression of maintenance, locomotive, or SB (Mason and Rushen, 2006). Although their maintenance level was higher, locomotion, cage shaking, and SB were lower than among cynomolgus individuals. Could it be that the two species differently respond to suboptimal conditions: rhesus macaques tend to display a passive response while cynomolgus monkeys perseverate actively? If that was the case, rhesus macaques would be more susceptible to a despair state since inactivity is the key criteria in most animal models of MDD (O'Neil and Moore, 2003; Nestler and Hyman, 2010). In the social-housed animals, these differences were reversed which might partly be associated with the inter-facility differences (see next paragraph). The higher rate of slumped posture and body facing the wall in both housing conditions, as well as the higher rate of gazes directed at the still environment in single-housed individuals, might also lead toward the hypothesis of a passive coping response in non-depressive rhesus monkeys.

Relative to body postures, the rhesus social group displayed five times less “on bars” posture than cynomolgus monkeys, which might be linked to the ability of the latter to move along arboreal pathways in the wild while rhesus are more terrestrial (Roonwal and Mohnot, 1977; Sussman and Tattersall, 1981; Roder and Timmermans, 2002).

The locations of the animals within the cage differed between the species. While the “up” and “bottom” differences were likely associated with the “on bars” and “seated” differences in single-housed monkeys, the cage spatial occupation might be associated with hierarchical ranks in socially-housed macaques. Thierry et al. described 4 grades of social hierarchies among macaque species from highly strict/nepotistic (grade 1) to highly permissive/tolerant (grade 4) (Thierry et al., 2000). Rhesus monkeys belong to grade 1 and cynomolgus monkeys to grade 2. The preferred sitting spots might therefore be restricted to high ranking individuals while low ranking animals are compelled to stay on the ground or on bars in the rhesus group. On the contrary among the cynomolgus animals the best sitting areas were more accessible to every member of the group. The same hypothesis can be raised from the inter-peer distances, since rhesus monkeys stood at a larger distance from their peers relative to cynomolgus, maybe to avoid aggressions and increase successful flights more frequent in the stricter grade 1 hierarchy. Although literature on inter-individual distances is not abundant, a link has been made between the strictness of the hierarchy and this feature with members of more tolerant hierarchies standing closer to their peer than members of strict hierarchies (de Waal and Luttrell, 1989; Zhang and Watanabe, 2007).

A few **inter-facility housing and breeding processes** might have accounted for a few significant differences as well, especially in the outdoor socially-housed monkeys.

For instance, the reduced feeding time among rhesus was likely due to the location of the feeding tray in the unattainable indoor part of the cage (whose access was blocked during observations) while it was in the observation cage in the cynomolgus groups (observations took place outside the feeding times but some food still remained in the tray during data collection). The lack of access to the feeding tray might also explain the higher rate of exploration (trying to find food) in the rhesus group. Their lower expression of resting behaviors might be a seasonal effect. Indeed the cynomolgus group was observed in Southern China in April (high humidity level and temperature) while the rhesus monkeys were observed in Northern China in October (lower humidity level and lower temperature). An ecological study reported a decrease of rest in the winter/dry season compared to the monsoon and summer seasons among commensal urban rhesus macaques (Jaman and Huffman, 2013).

Finally, some of the inter-species differences among the non-depressive groups might be biased by the fact that we pooled several clusters of individuals. Indeed their discrimination might be associated with several factors that have been shown to influence behavioral differences among non-human primate populations, e.g., dominance rank, temperament, age, parturition number, density, early experience (Stevenson-Hinde et al., 1980; de Waal and Luttrell, 1989; Veenema et al., 1997; Berard, 1999; de Waal et al., 2000; Thierry et al., 2000; Zhang and Watanabe, 2007; Freeman and Gosling, 2010). Age and parturition number were indeed correlated with several behavioral features in the non-depressive socially-housed cynomolgus monkeys. These questions have been discussed in our previous paper (Camus et al., 2013b) and will not be repeated here. Further data exploitation of the multiple correspondence analyses (e.g., contributions of each modality to the different factors) and/or further analyses of the collected data would have been required to properly investigate the features underlying the distinction between the non-pathological clusters. Yet, our aim was rather to focus on the atypical ones.

#### Inter-species differences in depressive-like animals

Interestingly, in both housing conditions, very few significant differences were reported between rhesus and cynomolgus **depressive-like** monkeys. This was especially striking in single-housed animals which differed only on the seated/bottom and on bars/up parameters. In the social housing condition, a few behaviors, body orientations and inter-peer distances differed relative to species. However, in both conditions, these parameters also differed among the non-depressive animals (and have been discussed in the previous section), suggesting an effect due to species-specific ecology or breeding processes rather than distinct depressive-like profiles. These results suggest that, in both rhesus and cynomolgus populations, the few individuals, which do not cope well with the captive housing conditions, respond in a rather identical way, i.e., similar depressive-like symptoms are developed whatever the species.

Despite the similar behavioral depressive-like features, the prevalence of such profiles differed between the two species. In social housing, 17.14% (6 out of 35 screened animals) of the rhesus and 6.25% [5 out of 80 screened animals (Camus et al., 2013b)] of the cynomolgus macaques displayed depressive-like features. In the single-housing condition, 45% (18 out of 40 screened animals) of the rhesus and 10% [4 out of 40 screened animals (Camus et al., 2013a)] of the cynomolgus monkeys expressed such features. In the literature, 38–42% of socially-housed cynomolgus monkeys submitted to social reorganization (Shively and Willard, 2012), 60% of the mice submitted to chronic social defeat (Krishnan et al., 2007) and 60% of the rats submitted to a forced swim test-retest paradigm (Shishkina et al., 2010) presented specific depressive-like behaviors. Compared to these preclinical data, the prevalence of depressive-like profiles in our socially-housed monkeys was therefore more similar to the ones reported in women (i.e., around 12.9% Berton and Nestler, 2006; Haenisch and Bonisch, 2011). In single-housed monkeys, the prevalence was higher than in men (i.e., 7.7% Berton and Nestler, 2006; Haenisch and Bonisch, 2011), especially in the rhesus group. This is not surprising given the extreme sub-optimality of this housing condition that does not fulfill the ecological needs of macaques (i.e., a hierarchically organized multi-male multi-female group allowing social interactions), conversely to socially-housing, and can therefore not be compared with a common man population. A more appropriate comparison could be the prevalence of MDD in male prisons. Depending on the race, age and criminal history of the subjects, MDD has been reported in 10–32% of male prisoners, which is much closer to the prevalence we observed in our study (Fazel and Danesh, 2002; Hassan et al., 2011; Heffernan et al., 2012; Naidoo and Mkize, 2012). Matching the human occurrence rate of a disease (i.e., the “population validity”) would give additional face value to an animal model (Schmidt, 2011).

Although age and weight significantly differed between rhesus and cynomolgus depressive-like individuals, neither parameter correlated with the expression of behaviors, thereby minimizing their role in this distinct inter-species prevalence. Moreover, MDD is not restricted to adults and affects children and adolescent in similar ways (Rao and Chen, 2009; Maalouf et al., 2011).

Our results support the assumption (made above) that rhesus macaques might be better suited to study the “spontaneous” development of depressive-like symptoms. One explanation could be the differences in the dominance hierarchies between rhesus and cynomolgus societies (see the above discussion about grade 1 and 2 societies Thierry et al., 2000). Indeed theorization of the development of depressive symptoms in Humans involves the accumulation of stress throughout life, i.e., the cumulative stress hypothesis (Nederhof and Schmidt, 2012). Both species encountered approximately the same types of adverse events in the breeding farms (early weaning around 6 months old, peer-rearing afterwards and pre-shipment single-housing) but the stricter hierarchy among rhesus groups during the first 6 months of their lives might be the additional trigger increasing the risk for later development of a depressive-like state. It might therefore be more profitable to study depressive-like symptoms in rhesus monkeys.

## Limitations

In every experimental setting, a few parameters remain uncontrollable. In this study the following ones might have influenced our results. First, despite the habituation phase, the avoidance of direct stares and the lack of significant differences in the level of behaviors directed toward the observer among housing conditions or species, we cannot rule out the possibility that rhesus and cynomolgus macaques might be differentially affected by the observer's presence near the cages. Whenever possible, video monitoring should be considered over direct observations. Second, assessing hierarchical ranks in social-housing conditions would have helped us to answer some of our hypotheses. Therefore it is particularly important for future studies to assess this type of hierarchical index to improve the characterization of the behavioral profiles. Finally, as mentioned in the Materials and Methods section, due to breeding processes, the single-housed animals were all males whereas the socially-housed monkeys were females. Although data from single- and social- housing conditions were not compared in our study, the effects due to gender and/or housing cannot be dissociated with our data and would require further experiments.

## Conclusions

This study shows that depressive-like individuals can be identified using ethological observations of daily life behaviors and associated parameters in the home cage of male single- and female socially-housed captive rhesus macaques. Many species-specific behavioral features were identified between rhesus and cynomolgus non-depressive monkeys, though phylogenetically close. One should thus be cautious when choosing its model species, regarding its aim and the observable variables of interest. We reported similar depressive-like profiles in both species. However, the prevalence of these profiles suggest that rhesus might be more vulnerable to developing a despair-like state relative to cynomolgus macaques both in single- and in social- housing conditions, and therefore more suitable as a “spontaneous” model of depressive disorders.

## Financial support

This work was supported by Biothèque Primate—Centre National de la Recherche Scientifique Life Sciences Department (Erwan Bezard) and an unrestricted grant from Motac Neuroscience Ltd., UK (Erwan Bezard). The funders had no role in study design, data collection and analysis, decision to publish, or preparation of the manuscript. Erwan Bezard has equity stake in Motac Holding Ltd. Erwan Bezard and Qin Li receive consultancy payments from Motac Neuroscience Ltd. All other authors have declared that no conflict of interest exists. This does not alter our adherence to all the policies on sharing data and materials.

## Author contributions

All authors revised the manuscript, gave final approval and contributed in the following ways: Sandrine M. J. Camus, conception and design (CD), acquisition of data (AD), analysis (A), and interpretation (I) of data; Céline Rochais, AD; Catherine Blois-Heulin, CD, I; Qin Li, AD; Martine Hausberger, CD, I; and Erwan Bezard, CD, I.

### Conflict of interest statement

The authors declare that the research was conducted in the absence of any commercial or financial relationships that could be construed as a potential conflict of interest.

## Supplementary material

Table S1 **Rhesus and cynomolgus monkey behavioral repertoire**. Collected detailed items (adapted from Camus et al., 2013a,b) were grouped for multiple component analysis (MCA).

Table S2 **Locations, distances to nearest peer, body postures, and orientations items displayed by rhesus and cynomolgus monkeys**. Collected detailed items (adapted from Camus et al., 2013a,b) were grouped for multiple component analysis (MCA).

Table S3 **Mann-Whitney U statistics among single-housed monkeys**.

Table S4 **Mann-Whitney U statistics among socially-housed monkeys**.

Figure S1 **Individual occurrence percentages of a few behavioral and postural parameters in rhesus monkeys**. The percentages of occurrence with regards to the total number of scans were calculated for each collected variable in the 40 single- (**panel A**) and the 35 socially- (**panel B**) housed rhesus monkeys. Individual values of the depressive-like (red dots) and non-depressive (green dots) monkeys are reported for a selection of variables. These variables significantly differed between the depressive-like and non-depressive animals in both housing conditions (**bold** font) or only in the respective group (*italic* font). The black lines indicate the mean of the 40 or 35 individuals. “B,” “L,” and “G” stand for “behavior,” “location,” and “gaze.” See Tables S1, S2 for a detailed description of each variable.

Figure S2 **Dendrogram resulting from a hierarchical cluster analysis in single-housed rhesus monkeys**. Following the multiple component analysis, the coordinates of the 40 single-housed rhesus monkeys were submitted to a hierarchical cluster analysis. The level indexes (above 0.10) are indicated on the dendrogram. One partition (blue arrow) resulted in 9 clusters for which the intra-cluster inertias are indicated on the right side of the graph. Animals from clusters 1, 2, and 8 (red font) expressed behavioral features similar to a few depressive symptoms whereas animals from the other clusters (green font) did not.

Figure S3 **Dendrogram resulting from a hierarchical cluster analysis in socially-housed rhesus monkeys**. Following the multiple component analysis, the coordinates of the 35 socially-housed rhesus monkeys were submitted to a hierarchical cluster analysis. The level indexes (above 0.10) are indicated on the dendrogram. One partition (blue arrow) resulted in 8 clusters for which the intra-cluster inertias are indicated on the right side of the graph. Animals from clusters 1, 4, and 7 (red font) expressed behavioral features similar to a few depressive symptoms whereas animals from the other clusters (green font) did not.

## References

[B1] Altmann J. (1974). Observational study of behavior: sampling methods. Behaviour 49, 227–267 10.1163/156853974X00534 4597405

[B2] American Psychiatric Association. (1994). Diagnostic and Statistical Manual of Mental Disorders Washington, DC: American Psychiatric Press

[B3] Berard J. (1999). A four-year study of the association between male dominance rank, residency status, and reproductive activity in rhesus macaques (*Macaca mulatta*). Primates 40, 159–175 10.1007/BF02557708 23179538

[B4] Berton O. Hahn C. G. Thase M. E. (2012). Are we getting closer to valid translational models for major depression? Science 338, 75–79 10.1126/science.1222940 23042886

[B5] Berton O. Nestler E. J. (2006). New approaches to antidepressant drug discovery: beyond monoamines. Nat. Rev. Neurosci. 7, 137–151 10.1038/nrn1846 16429123

[B6] Camus S. M. J. Blois-Heulin C. Li Q. Hausberger M. Bezard E. (2013a). Behavioural profiles in captive-bred cynomolgus macaques: towards monkey models of mental disorders? PLoS ONE 8:e62141 10.1371/journal.pone.0062141 23658620 PMC3639229

[B7] Camus S. M. J. Rochais C. Blois-Heulin C. Li Q. Hausberger M. Bezard E. (2013b). Birth origin differentially affects depressive-like behaviours: are captive-born cynomolgus monkeys more vulnerable to depression than their wild-born counterparts? PLoS ONE 8:e67711 10.1371/journal.pone.0067711 23861787 PMC3701588

[B8] Claessens S. E. F. Daskalakis N. P. van der Veen R. Oitzl M. S. de Kloet E. R. Champagne D. L. (2011). Development of individual differences in stress responsiveness: an overview of factors mediating the outcome of early life experiences. Psychopharmacology 214, 141–154 10.1007/s00213-010-2118-y 21165737 PMC3045508

[B9] Clarke A. S. Mason W. A. (1988). Differences among three Macaque species in responsiveness to an observer. Int. J. Primatol. 9, 347–368 10.1007/BF02737382

[B10] Crockett C. M. Bowers C. L. Shimoji M. Leu M. Bowden D. M. Sackett G. P. (1995). Behavioral responses of longtailed macaques to different cage sizes and common laboratory experiences. J. Comp. Psychol. 109, 368–383 10.1037/0735-7036.109.4.368 7497695

[B11] Crofts H. S. Muggleton N. G. Bowditch A. P. Pearce P. C. Nutt D. J. Scott E. A. (1999). Home cage presentation of complex discrimination tasks to marmosets and rhesus monkeys. Lab. Anim. 33, 207–214 10.1258/002367799780578174 10780838

[B12] Daskalakis N. P. Oitzl M. S. Schachinger H. Champagne D. L. de Kloet E. R. (2012). Testing the cumulative stress and mismatch hypotheses of psychopathology in a rat model of early-life adversity. Physiol. Behav. 106, 707–721 10.1016/j.physbeh.2012.01.015 22306534

[B13] de Kloet E. R. Joels M. Holsboer F. (2005). Stress and the brain: from adaptation to disease. Nat. Rev. Neurosci. 6, 463–475 10.1038/nrn1683 15891777

[B14] de Waal F. B. Aureli F. Judge P. G. (2000). Coping with crowding. Sci. Am. 282, 76–81 10.1038/scientificamerican0500-76 11056991

[B15] de Waal F. B. M. Luttrell L. M. (1989). Toward a comparative socioecology of the genus Macaca: different dominance styles in rhesus and stumptail monkeys. Am. J. Primatol. 19, 83–109 10.1002/ajp.1350190203 31964014

[B16] Fazel S. Danesh J. (2002). Serious mental disorder in 23000 prisoners: a systematic review of 62 surveys. Lancet 359, 545–550 10.1016/S0140-6736(02)07740-1 11867106

[B17] Fooden J. (2000). Systematic Review of the Rhesus Macaque, Macaca mulatta (Zimmermann, 1780). Fieldiana Zoology. (Chicago, IL: Field Museum of Natural History), 96

[B18] Freeman H. D. Gosling S. D. (2010). Personality in nonhuman primates: a review and evaluation of past research. Am. J. Primatol. 72, 653–671 10.1002/ajp.20833 20568079

[B19] Fureix C. Jego P. Henry S. Lansade L. Hausberger M. (2012). Towards an ethological animal model of depression? A study on horses. PLoS ONE 7:e39280 10.1371/journal.pone.0039280 22761752 PMC3386251

[B20] Geerts E. Brune M. (2009). Ethological approaches to psychiatric disorders: focus on depression and schizophrenia. Aust. N.Z. J. Psychiatry 43, 1007–1015 10.1080/00048670903270498 20001396

[B21] Gilbert M. H. Baker K. C. (2011). Social buffering in adult male rhesus macaques (*Macaca mulatta*): effects of stressful events in single vs pair housing. J. Med. Primatol. 40, 71–78 10.1111/j.1600-0684.2010.00447.x 21371035 PMC3058767

[B22] Haenisch B. Bonisch H. (2011). Depression and antidepressants: insights from knockout of dopamine, serotonin or noradrenaline re-uptake transporters. Pharmacol. Ther. 129, 352–368 10.1016/j.pharmthera.2010.12.002 21147164

[B23] Harlow H. F. Suomi S. J. (1974). Induced depression in monkeys. Behav. Biol. 12, 273–296 10.1016/S0091-6773(74)91475-8 4475586

[B24] Hassan L. Birmingham L. Harty M. A. Jarrett M. Jones P. King C. (2011). Prospective cohort study of mental health during imprisonment. Br. J. Psychiatry 198, 37–42 10.1192/bjp.bp.110.080333 21200075

[B25] Heffernan E. B. Andersen K. C. Dev A. Kinner S. (2012). Prevalence of mental illness among Aboriginal and Torres Strait Islander people in Queensland prisons. Med. J. Aust. 197, 37–41 10.5694/mja11.11352 22762230

[B26] Henry D. B. Tolan P. H. Gorman-Smith D. (2005). Cluster analysis in family psychology research. J. Fam. Psychol. 19, 121–132 10.1037/0893-3200.19.1.121 15796658

[B27] Jaman M. F. Huffman M. A. (2013). The effect of urban and rural habitats and resource type on activity budgets of commensal Rhesus macaques (*Macaca mulatta*) in Bangladesh. Primates 54, 49–59 10.1007/s10329-012-0330-6 22987063

[B28] Kalin N. H. Shelton S. E. (2003). Nonhuman primate models to study anxiety, emotion regulation, and psychopathology. Ann. N.Y. Acad. Sci. 1008, 189–200 10.1196/annals.1301.021 14998885

[B29] Krishnan V. Han M.-H. Graham D. L. Berton O. Renthal W. Russo S. J. (2007). Molecular adaptations underlying susceptibility and resistance to social defeat in brain reward regions. Cell 131, 391–404 10.1016/j.cell.2007.09.018 17956738

[B30] Maalouf F. T. Atwi M. Brent D. A. (2011). Treatment-resistant depression in adolescents: review and updates on clinical management. Depress. Anxiety 28, 946–954 10.1002/da.20884 21898710

[B31] Mason G. J. Rushen J. (2006). Sterotypic Animal Behaviour: Fundamentals and Applications to Welfare. Wallingford: CABI 10.1079/9780851990040.0000

[B32] Matthews K. Christmas D. Swan J. Sorrell E. (2005). Animal models of depression: navigating through the clinical fog. Neurosci. Biobehav. Rev. 29, 503–513 10.1016/j.neubiorev.2005.03.005 15925695

[B33] Montaudouin S. Le Pape G. (2004). Comparison of the behaviour of European brown bears (*Ursus arctos* arctos) in six different parks, with particular attention to stereotypies. Behav. Processes 67, 235–244 10.1016/j.beproc.2004.02.008 15497257

[B34] Naidoo S. Mkize D. L. (2012). Prevalence of mental disorders in a prison population in Durban, South Africa. Afr. J. Psychiatry (Johannesbg.) 15, 30–35 10.4314/ajpsy.v15i1.4 22344760

[B35] Nederhof E. Schmidt M. V. (2012). Mismatch or cumulative stress: toward an integrated hypothesis of programming effects. Physiol. Behav. 106, 691–700 10.1016/j.physbeh.2011.12.008 22210393

[B36] Nestler E. J. Hyman S. E. (2010). Animal models of neuropsychiatric disorders. Nat. Neurosci. 13, 1161–1169 10.1038/nn.2647 20877280 PMC3750731

[B37] O'Neil M. F. Moore N. A. (2003). Animal models of depression: are there any? Hum. Psychopharmacol. 18, 239–254 10.1002/hup.496 12766928

[B38] Post W. Baulu J. (1978). Time budgets of *Macaca mulatta*. Primates 19, 125–140 10.1007/BF02373230

[B39] Rao U. Chen L. A. (2009). Characteristics, correlates, and outcomes of childhood and adolescent depressive disorders. Dialogues Clin. Neurosci. 11, 45–62 19432387 10.31887/DCNS.2009.11.1/urao PMC2766280

[B40] Roder E. L. Timmermans P. J. (2002). Housing and care of monkeys and apes in laboratories: adaptations allowing essential species-specific behaviour. Lab. Anim. 36, 221–242 10.1258/002367702320162360 12144737

[B41] Roonwal M. L. Mohnot S. M. (1977). Primates of South Asia: Ecology, Sociobiology, and Behavior. Cambridge, MA: Harvard University Press

[B42] Schino G. Perretta G. Taglioni A. M. Monaco V. Troisi A. (1996). Primate displacement activities as an ethopharmacological model of anxiety. Anxiety 2, 186–191 10.1002/(SICI)1522-7154(1996)2:4<186::AID-ANXI5>3.0.CO;2-M 9160621

[B43] Schmidt M. V. (2011). Animal models for depression and the mismatch hypothesis of disease. Psychoneuroendocrinology 36, 330–338 10.1016/j.psyneuen.2010.07.001 20674180

[B44] Seay B. Gottfried N. W. (1975). A phylogenetic perspective for social behavior in primates. J. Gen. Psychol. 92, 5–17 10.1080/00221309.1975.9711323 803553

[B45] Shishkina G. T. Kalinina T. S. Berezova I. V. Bulygina V. V. Dygalo N. N. (2010). Resistance to the development of stress-induced behavioral despair in the forced swim test associated with elevated hippocampal Bcl-xl expression. Behav. Brain Res. 213, 218–224 10.1016/j.bbr.2010.05.003 20457187

[B46] Shively C. A. Willard S. L. (2012). Behavioral and neurobiological characteristics of social stress versus depression in nonhuman primates. Exp. Neurol. 233, 87–94 10.1016/j.expneurol.2011.09.026 21983263 PMC4031682

[B47] Stevenson-Hinde J. Stillwell-Barnes R. Zunz M. (1980). Individual differences in young rhesus monkeys: consistency and change. Primates 21, 498–509 10.1007/BF02373838

[B48] Suomi S. J. Eisele C. D. Grady S. A. Harlow H. F. (1975). Depressive behavior in adult monkeys following separation from family environment. J. Abnorm. Psychol. 84, 576–578 10.1037/h0077066 811700

[B49] Sussman A. F. Ha J. C. Bentson K. L. Crockett C. M. (2013). Temperament in rhesus, long-tailed, and pigtailed macaques varies by species and sex. Am. J. Primatol. 75, 303–313 10.1002/ajp.22104 23225368 PMC3581757

[B50] Sussman R. Tattersall I. (1981). Behavior and ecology of *Macaca fascicularis* in Mauritius: a preliminary study. Primates 22, 192–205 10.1007/BF02382610

[B51] Thierry B. Iwaniuk A. N. Pellis S. M. (2000). The influence of phylogeny on the social behaviour of macaques. Ethology 106, 713–728 10.1046/j.1439-0310.2000.00583.x

[B52] Tinbergen N. (1952). Derived activities: their causation, biological significance, origin, and emancipation during evolution. Q. Rev. Biol. 27, 1–32 10.1086/398642 14930222

[B53] Troisi A. (2002). Displacement activities as a behavioral measure of stress in nonhuman primates and human subjects. Stress 5, 47–54 10.1080/102538902900012378 12171766

[B54] Veenema H. C. Spruijt B. M. Gispen W. H. van Hooff J. A. (1997). Aging, dominance history, and social behavior in Java-monkeys (*Macaca fascicularis*). Neurobiol. Aging 18, 509–515 10.1016/S0197-4580(97)00107-3 9390777

[B55] Willner P. (1997). Validity, reliability and utility of the chronic mild stress model of depression: a 10-year review and evaluation. Psychopharmacology (Berl.) 134, 319–329 10.1007/s002130050456 9452163

[B56] Zhang P. Watanabe K. (2007). Extra-large cluster formation by Japanese macaques (*Macaca fuscata*) on Shodoshima Island, central Japan, and related factors. Am. J. Primatol. 69, 1119–1130 10.1002/ajp.20419 17330869

