# Supplementary material for: Depressive-like behavioral profiles in captive-bred single- and socially-housed rhesus and cynomolgus macaques: a species comparison
Source: Front Behav Neurosci. 2014 Feb 19;8:47. doi: 10.3389/fnbeh.2014.00047 (PMC3928569; doi:10.3389/fnbeh.2014.00047)
Supplement: Table S1 — Rhesus and cynomolgus monkey behavioral repertoire. Collected detailed items (adapted from Camus et al., 2013a,b) were grouped for multiple component analysis (MCA). [file DataSheet1.DOCX]

Table S1 : Rhesus and cynomolgus monkey behavioural repertoire.

| **Grouped behaviours for MCA** | **Detailed collected behaviours** |
| --- | --- |
| **displacement behaviours** | scratches self |
|  | yawns |
|  | vacuum chew (chews despite empty mouth and cheekpouches) |
| **feeding** | "hunts" insect (try or manage to catch) |
|  | eats or drinks |
| **behaviours towards human** | interacts with observer (threat, submission, lipsmacking, genital display) |
| **inactivity** | immobility: not engaged in any other behaviours, with open eyes |
|  | rests: not engaged in any other behaviours, with closing or closed eyes |
|  | tensed inactivity (usually after an aggressive encounter) |
| **investigation** | manipulates collar with hands and/or mouth non-repetitively |
|  | manipulates toy with hands and/or mouth non-repetitively |
|  | manipulates other object with hands and/or mouth non-repetitively |
|  | cage investigation (searches, sniffs wall or bars) |
|  | uses feeding tray as a mirror, looks into it |
| **locomotion** | locomotion : change of location without any other behaviours |
|  | dangles on the swing |
| **maternal behaviours** | grooms infant or displays affiliative facial expression - positive |
|  | rejects, threatens, chases, bites or slapes infant - negative |
|  | retrieves or restraints infant - restrictive |
| **maintenance behaviours** | selfgrooms: grooming of own body |
|  | maintenance behaviours (urinate, defecate, rub eyes) |
|  | bites its nails |
|  | rubs hands on bars or floor |
| **social behaviours** | grooms peer- affiliative |
|  | is groomed by peer - affiliative |
|  | presents body part to be groomed - affiliative |
|  | presents genitals in a non-sexual context - affiliative |
|  | lipsmacks – affiliative or submissive |
|  | other affiliative behaviours (facial expression, seak positive contact with peer) |
|  | threatens, chases, bites or slapes peer - agonistic |
|  | flees or displays submissive facial expression - submissive |
| **sexual behaviours** | typical sexual behaviour (presents genitals to male, mates) |
| **vocalization** | vocalizes |
| **stereotypic behaviours** | manipulates collar repetitively - manual |
|  | manipulates other object repetitively - manual |
|  | picks peer’s or own fur repetitively and outside grooming context - manual |
|  | motor stereotypy (pacing, flipping, walks backwards,…) - motor |
|  | gnaws bars repetitively - oral |
|  | licks bars repetitively - oral |
|  | licks own tail or other body part repetitively - oral |
|  | bites own tail or other body part repetitively - oral |
|  | oral stereotypy (tongue movement or tongue chew) - oral |
|  | steals infant from its mother - manual |
|  | self suckling - oral |

**Collected detailed items (adapted from (**[**Camus et al., 2013a**](#_ENREF_6)**,** [**Camus et al., 2013b**](#_ENREF_7)**)) were grouped for multiple component analysis (MCA).**
