# Supplementary material for: Depressive-like behavioral profiles in captive-bred single- and socially-housed rhesus and cynomolgus macaques: a species comparison
Source: Front Behav Neurosci. 2014 Feb 19;8:47. doi: 10.3389/fnbeh.2014.00047 (PMC3928569; doi:10.3389/fnbeh.2014.00047)
Supplement: Table S2 — Locations, distances to nearest peer, body postures, and orientations items displayed by rhesus and cynomolgus monkeys. Collected detailed items (adapted from Camus et al., 2013a,b) were grouped for multiple component analysis (MCA). [file DataSheet2.DOCX]

Table S2. Locations, distances to nearest peer, body postures and orientations items displayed by rhesus and cynomolgus monkeys.

| **Variables for MCA** | **Detailed collected variables** |
| --- | --- |
| **Body postures** |  |
| **seated** | resting on the buttocks with straight back |
|  | resting on the buttocks with bent back |
|  | resting on the buttocks with stretched legs |
| **biped** | standing on hind limbs (biped) |
| **slumped** | slumped (seated head lower than shoulder's line) |
| **lying down** | lying down |
| **on bars** | seated posture but on on a wire mesh part of the cage |
|  | upside down four-legged (hanging on wire meshed ceiling) |
|  | suspending in any other way (four limbs on a wire meshed part of the cage) |
| **four-legged** | four-legged, hanging tail |
|  | four-legged, tail in "?" shape |
|  | four-legged, tail above head |
|  | four-legged, straight tail (in the continuity of the back) |
|  | « bottom up » (four-legged with head and shoulders on the ground level) |
|  | crouched (ventral surface close to floor; head at or below the level of the shoulders) |
|  |  |
| **Body orientations** |  |
| **peer** | body oriented toward peer **(in social-housing only)** |
| **exterior** | body oriented toward exterior/observer |
| **ground** | body oriented toward ground or ceiling |
| **wall** | body oriented toward a wall of the cage (max. 50 cm from wall) |
| **open environment** | body oriented toward open environment (none of the above) **(in social-housing only)** |
|  |  |
| **Locations in cage** |  |
| **side** | cage width divided in 3 virtual parts: side or middle |
| **middle** |  |
| **front** | cage depth divided in 3 or **2** virtual parts: front, **(middle)** or back in social- or **single-**housing |
| **middle** |  |
| **back** |  |
| **bottom** | cage height divided in 3 or 2 virtual parts: up, **(sitting bench)** or bottom in social- or **single**-housing |
| **sitting bench** |  |
| **up** |  |
|  |  |
| **Gazes** | ***In single-housing only*** |
| **observer** | observer |
| **still environment** | wall |
|  | ground or ceiling |
| **object/self** | manipulable object (feeding or water tray, cage lock) |
|  | own body |
| **living environment** | peer |
|  | outside |
|  | insect |
|  |  |
| **Distance to nearest peer** | ***In social-housing only*** |
| **against** | body in direct physical contact with a peer |
| **d. < 1arm** | within 1 arm from peer |
| **1arm<d.<1m** | between 1 arm and 1 meter from peer |
| **1m<d.<3m** | between 1 meter and 3 meter from peer |
| **d.>3m** | more than 3 meter from peer |

**Collected detailed items (adapted from (**[**Camus et al., 2013a**](#_ENREF_6)**,** [**Camus et al., 2013b**](#_ENREF_7)**)) were grouped for multiple component analysis (MCA).**
