# Supplementary material for: Depressive-like behavioral profiles in captive-bred single- and socially-housed rhesus and cynomolgus macaques: a species comparison
Source: Front Behav Neurosci. 2014 Feb 19;8:47. doi: 10.3389/fnbeh.2014.00047 (PMC3928569; doi:10.3389/fnbeh.2014.00047)
Supplement: Table S3 — Mann-Whitney U statistics among single-housed monkeys. [file DataSheet3.DOCX]

Table S3. Mann-Whitney U statistics among single-housed monkeys.

| **MW U test statistics** | | **Rhe vs cyno**  **total**  **‡ 1** | **Depr vs ND**  **rhesus**  **†** | **Depr vs ND**  **cyno**  **†** | **Cyno vs rhe**  **depr**  **‡ 2** | **Cyno vs rhe**  **ND**  **‡ 3** |
| --- | --- | --- | --- | --- | --- | --- |
|  | **weight (kg)** | 0.0 | 93.0 | 54.5 | 0.0 | 0.0 |
|  | **age (years old)** | 201.0 | 183.0 | 55.5 | 15.5 | 48.0 |
| **Behaviours** | **displacement B.** | 253.0 | 101.5 | 47.0 | 14.5 | 166.0 |
|  | **scratch** | 300.5 | 134.5 | 36.5 | 18.5 | 162.0 |
|  | **vacuous chew** | 408.5 | 160.5 | 58.0 | 13.0 | 245.0 |
|  | **yawn** | 427.0 | 135.0 | 57.0 | 21.0 | 242.5 |
|  | **feeding B.** | 450.0 | 129.0 | 23.0 | 31.0 | 226.5 |
|  | **B. toward human** | 713.0 | 97.5 | 38.0 | 26.0 | 218.0 |
|  | **threat** | 677.5 | 104.5 | 46.0 | 28.0 | 203.5 |
|  | **submission** | 799.5 | 180.5 | 58.0 | 34.0 | 357.0 |
|  | **inactivity** | 303.5 | 2.5 | 0.0 | 29.0 | 174.0 |
|  | **immobility** | 323.0 | 17.0 | 0.0 | 34.0 | 177.0 |
|  | **resting B.** | 492.0 | 121.5 | 57.0 | 22.0 | 274.5 |
|  | **investigation** | 387.5 | 39.0 | 3.0 | 29.0 | 218.5 |
|  | **locomotion** | 502.0 | 171.5 | 20.5 | 30.5 | 209.0 |
|  | **maintenance B.** | 689.0 | 38.5 | 34.5 | 32.0 | 144.5 |
|  | **sexual B.** | 681.5 | 183.5 | 65.0 | 29.5 | 333.5 |
|  | **shake** | 266.0 | 144.0 | 21.5 | 27.0 | 101.0 |
|  | **social B.** | 287.0 | 118.0 | 24.0 | 21.0 | 139.5 |
|  | **allogrooming** | 0.0 | 198.0 | 44.0 | 36.0 | 242.0 |
|  | **stereotypic B.** | 370.5 | 97.5 | 14.5 | 18.0 | 240.5 |
|  | ***Behavioural switch*** | 243.0 | 18.5 | 0.0 | 22.0 | 122.0 |
|  | ***Behavioural diversity*** | 95.5 | 46.5 | 0.0 | 15.5 | 9.0 |
| **Body postures** | **biped** | 759.0 | 128.0 | 6.0 | 16.0 | 361.0 |
|  | **four-legged** | 500.5 | 70.5 | 6.5 | 29.5 | 312.5 |
|  | **lying** | 727.0 | 127.5 | 34.0 | 28.0 | 274.0 |
|  | **on bars** | 160.5 | 195.5 | 0.0 | 2.0 | 66.5 |
|  | **seated** | 599.0 | 71.0 | 8.0 | 1.0 | 300.0 |
|  | **slumped** | 453.5 | 87.5 | 57.0 | 29.0 | 104.5 |
| ***Main B. while slumped:*** | |  |  |  |  |  |
|  | **inactivity** | 471.0 | 89.5 | 4.0 | 8.0 | 280.0 |
|  | **investigation** | 550.0 | 94.5 | 15.0 | 10.0 | 267.0 |
|  | **maintenance** | 471.5 | 103.5 | 19.5 | 10.0 | 215.0 |
|  | **social B.** | 0.0 | 136.5 | 27.0 | 13.0 | 283.5 |
| **Body orientations** | **outside** | 363.5 | 97.0 | 53.5 | 17.0 | 203.5 |
|  | **ground** | 590.0 | 76.0 | 26.5 | 29.0 | 359.0 |
|  | **wall** | 372.5 | 75.0 | 52.0 | 16.0 | 231.5 |
|  | ***B. while facing wall:*** |  |  |  |  |  |
|  | **feeding B.** | 672.0 | 191.0 | 36.0 | 28.0 | 289.0 |
|  | **inactivity** | 263.5 | 23.5 | 0.0 | 34.0 | 138.0 |
|  | **investigation** | 433.0 | 39.5 | 5.0 | 33.0 | 257.5 |
|  | **maintenance** | 693.0 | 52.0 | 36.0 | 36.0 | 193.5 |
|  | **social B.** | 472.5 | 175.0 | 28.0 | 34.0 | 182.0 |
| **Locations** | **front** | 361.5 | 61.5 | 16.5 | 24.0 | 219.0 |
|  | **back** | 362.5 | 61.5 | 16.5 | 24.0 | 220.0 |
|  | **bottom** | 281.5 | 167.0 | 3.0 | 2.0 | 130.5 |
|  | **up** | 281.0 | 167.0 | 3.0 | 2.0 | 130.0 |
|  | **middle** | 403.5 | 36.0 | 29.0 | 19.0 | 285.5 |
|  | **side** | 402.0 | 36.0 | 29.0 | 20.0 | 284.0 |
| **Gazes** | **still env.** | 432.5 | 138.5 | 41.5 | 28.0 | 227.5 |
|  | **wall** | 510.0 | 126.0 | 57.5 | 22.5 | 297.0 |
|  | **living env.** | 515.0 | 168.0 | 60.5 | 19.0 | 257.0 |
|  | **manipulable object** | 701.0 | 35.0 | 10.5 | 30.5 | 279.5 |
|  | **observer** | 601.5 | 151.5 | 54.0 | 32.5 | 230.0 |

**Abbreviations: cynomolgus (cyno), depressive-like (depr), non-depressive (ND), behaviour (B), environment (env), Mann-Whitney (MW).**
