# Supplementary material for: Depressive-like behavioral profiles in captive-bred single- and socially-housed rhesus and cynomolgus macaques: a species comparison
Source: Front Behav Neurosci. 2014 Feb 19;8:47. doi: 10.3389/fnbeh.2014.00047 (PMC3928569; doi:10.3389/fnbeh.2014.00047)
Supplement: Table S4 — Mann-Whitney U statistics among socially-housed monkeys. [file DataSheet4.DOCX]

Table S4. Mann-Whitney U statistics among socially-housed monkeys.

| **MW U test statistics** | | **Rhe vs cyno**  **Total ‡ 1** | **Depr vs ND**  **Rhesus †** | **Depr vs ND**  **Cyno †** | **Cyno vs rhe**  **Depr ‡ 2** | **Cyno vs rhe**  **ND ‡ 3** |
| --- | --- | --- | --- | --- | --- | --- |
|  | **weight (kg)** | 1.0 | 68.5 | 156.0 | 0.0 | 1.0 |
|  | **age (years old)** | 319.5 | 85.5 | 107.0 | 0.0 | 269.0 |
|  | **parturition number** | 988.0 | 73.5 | 77.5 | 4.5 | 770.0 |
| **Behaviours** | **displacement B.** | 708.0 | 62.5 | 83.0 | 3.5 | 538.0 |
|  | **scratch** | 637.0 | 65.5 | 92.5 | 2.5 | 491.0 |
|  | **vacuous chew** | 1380.0 | 82.0 | 122.5 | 10.0 | 1082.5 |
|  | **yawn** | 852.0 | 57.0 | 177.5 | 15.0 | 580.0 |
|  | **feeding B.** | 464.0 | 58.5 | 123.0 | 0.0 | 403.0 |
|  | **B. toward human** | 1389.5 | 78.0 | 167.0 | 12.0 | 1062.5 |
|  | **threat** | 1351.5 | 75.0 | 170.0 | 15.0 | 1028.0 |
|  | **submission** | 1298.0 | 66.0 | 172.5 | 10.0 | 1059.0 |
|  | **inactivity** | 1161.0 | 19.0 | 25.0 | 8.0 | 790.0 |
|  | **immobility** | 1193.0 | 74.5 | 81.0 | 10.0 | 887.0 |
|  | **resting B.** | 932.0 | 34.0 | 96.5 | 14.0 | 592.0 |
|  | **investigation** | 903.0 | 40.5 | 103.0 | 2.0 | 663.0 |
|  | **locomotion** | 1204.0 | 39.5 | 37.5 | 12.0 | 991.0 |
|  | **maternal** | 1344.0 | 78.0 | 88.0 | 7.0 | 1052.0 |
|  | **maintenance B.** | 896.0 | 42.0 | 97.5 | 6.0 | 641.0 |
|  | **sexual B.** | 1123.0 | 78.0 | 127.5 | 15.0 | 875.0 |
|  | **shake** | 1377.0 | 84.0 | 185.0 | 15.0 | 1064.0 |
|  | **social B.** | 1363.0 | 16.5 | 73.0 | 15.0 | 1003.0 |
|  | **allogrooming** | 938.0 | 21.5 | 111.0 | 13.0 | 566.0 |
|  | **stereotypic B.** | 1165.0 | 83.5 | 184.5 | 12.5 | 909.0 |
|  | ***Behavioural diversity*** | 1229.0 | 31.0 | 55.0 | 12.0 | 1002.0 |
| **Body postures** | **biped** | 1337.0 | 54.0 | 130.0 | 14.0 | 970.0 |
|  | **four-legged** | 933.0 | 26.0 | 31.5 | 9.0 | 790.0 |
|  | **lying** | 794.0 | 81.0 | 126.0 | 6.0 | 625.0 |
|  | **on bars** | 569.0 | 52.5 | 163.5 | 8.5 | 391.0 |
|  | **seated** | 391.0 | 80.5 | 173.5 | 5.0 | 289.0 |
|  | **slumped** | 593.0 | 78.5 | 88.0 | 11.0 | 432.0 |
|  | ***Main B. while slumped:*** |  |  |  |  |  |
|  | **inactivity** | 117.0 | 19.0 | 73.0 | 0.0 | 51.0 |
|  | **investigation** | 0.0 | 80.5 | 165.0 | 7.5 | 561.0 |
|  | **maintenance** | 109.5 | 46.0 | 115.0 | 2.5 | 48.0 |
|  | **social B.** | 576.0 | 34.0 | 105.5 | 2.0 | 422.5 |
| **Body orientations** | **peer** | 1070.0 | 75.0 | 177.0 | 11.0 | 851.0 |
|  | **outside** | 1179.0 | 77.5 | 33.0 | 1.0 | 834.0 |
|  | **ground** | 1369.0 | 47.5 | 37.0 | 15.0 | 1007.0 |
|  | **environment** | 1171.0 | 39.0 | 129.0 | 13.0 | 930.0 |
|  | **wall** | 711.0 | 84.5 | 124.0 | 6.0 | 572.0 |
|  | ***B. while facing wall:*** |  |  |  |  |  |
|  | **feeding B.** | 549.5 | 63.5 | 147.0 | 8.0 | 429.0 |
|  | **inactivity** | 1215.5 | 38.0 | 73.0 | 11.5 | 867.0 |
|  | **investigation** | 656.0 | 55.5 | 102.0 | 1.0 | 495.5 |
|  | **maintenance** | 1290.5 | 38.0 | 142.5 | 14.0 | 1068.0 |
|  | **social B.** | 1049.5 | 78.0 | 182.0 | 12.0 | 822.5 |
| **Locations** | **front** | 1227.5 | 72.5 | 109.0 | 14.0 | 976.5 |
|  | **back** | 680.0 | 78.5 | 55.0 | 4.0 | 522.0 |
|  | **bottom** | 839.5 | 82.0 | 73.0 | 11.0 | 575.5 |
|  | **sitting bench** | 669.0 | 79.0 | 77.0 | 12.0 | 453.0 |
|  | **up** | 605.0 | 56.0 | 179.0 | 10.0 | 423.0 |
|  | **middle** | 575.5 | 17.0 | 165.0 | 6.0 | 520.5 |
|  | **side** | 576.0 | 17.0 | 165.0 | 6.0 | 521.0 |
| **Distances** | **against** | 350.5 | 76.0 | 26.0 | 0.0 | 289.5 |
|  | **d. < 1arm** | 909.0 | 64.5 | 0.0 | 2.0 | 688.0 |
|  | **1arm<d.<1m** | 785.0 | 82.0 | 15.0 | 0.0 | 664.0 |
|  | **1m<d.<3m** | 784.0 | 69.0 | 141.0 | 5.0 | 636.0 |
|  | **d.>3m** | 984.0 | 86.0 | 148.0 | 15.0 | 720.5 |

**Abbreviations: cynomolgus (cyno), depressive-like (depr), non-depressive (ND), behaviour (B), distance (d), Mann-Whitney (MW).**
