# Supplementary material for: Depressive-like behavioral profiles in captive-bred single- and socially-housed rhesus and cynomolgus macaques: a species comparison
Source: Front Behav Neurosci. 2014 Feb 19;8:47. doi: 10.3389/fnbeh.2014.00047 (PMC3928569; doi:10.3389/fnbeh.2014.00047)

**Figure S1. Individual occurrence percentages of a few behavioural and postural parameters in rhesus monkeys.**

The percentages of occurrence with regards to the total number of scans were calculated for each collected variable in the 40 single- (**panel A**) and the 35 socially- (**panel B**) housed rhesus monkeys. Individual values of the depressive-like (red dots) and non-depressive (green dots) monkeys are reported for a selection of variables. These variables significantly differed between the depressive-like and non-depressive animals in both housing conditions (**bold** font) or only in the respective group (*italic* font). The black lines indicate the mean of the 40 or 35 individuals. “B”, “L” and “G” stand for “behaviour”, “location” and “gaze”. See **Tables** S**1 and S2** for a detailed description of each variable.


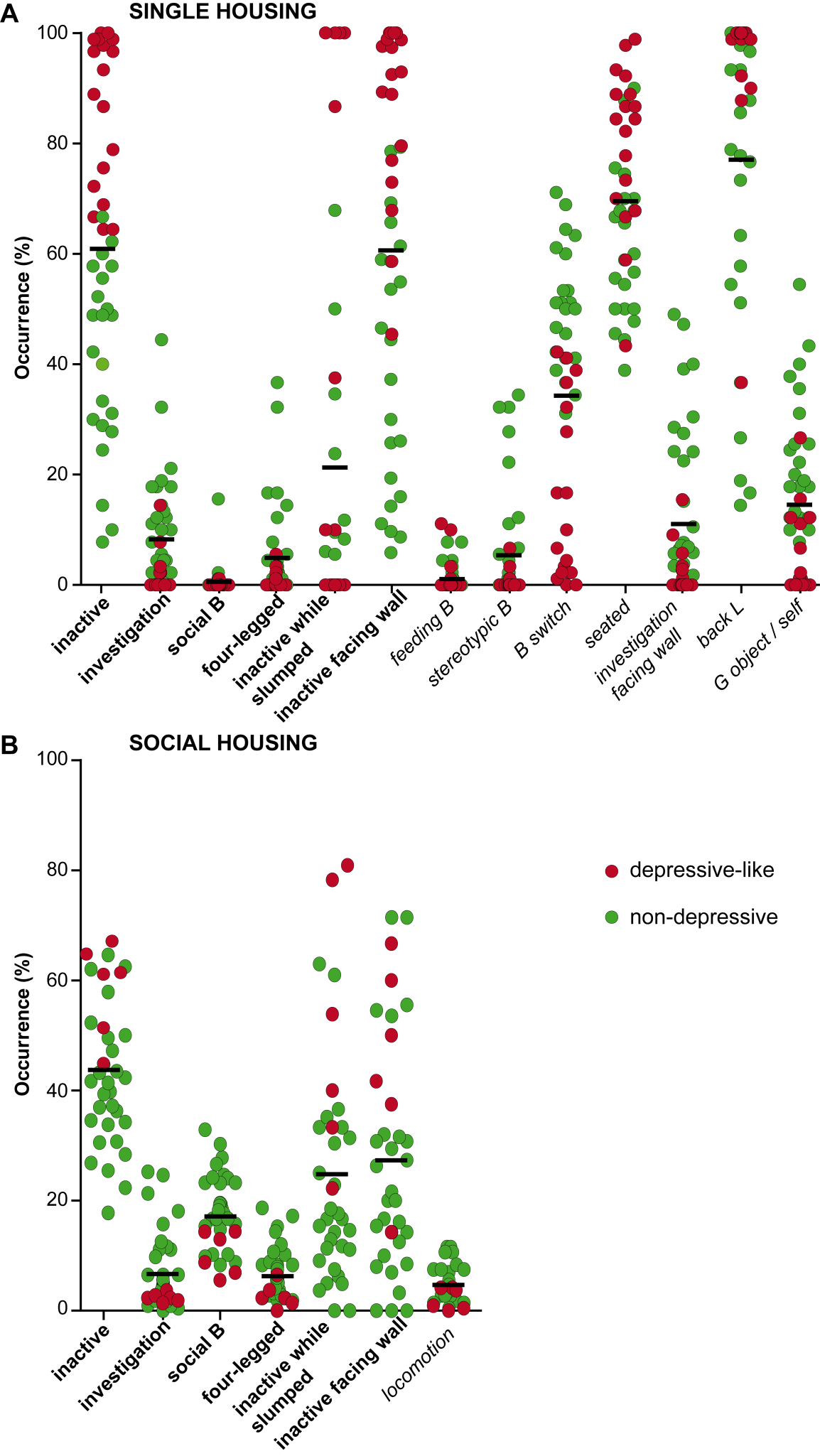

Supplement: Figure S1 — Individual occurrence percentages of a few behavioral and postural parameters in rhesus monkeys. The percentages of occurrence with regards to the total number of scans were calculated for each collected variable in the 40 single- (panel A) and the 35 socially- (panel B) housed rhesus monkeys. Individual values of the depressive-like (red dots) and non-depressive (green dots) monkeys are reported for a selection of variables. These variables significantly differed between the depressive-like and non-depressive animals in both housing conditions (bold font) or only in the respective group (italic font). The black lines indicate the mean of the 40 or 35 individuals. “B,” “L,” and “G” stand for “behavior,” “location,” and “gaze.” See Tables S1, S2 for a detailed description of each variable. [file DataSheet5.DOCX]
