# Supplementary material for: Depressive-like behavioral profiles in captive-bred single- and socially-housed rhesus and cynomolgus macaques: a species comparison
Source: Front Behav Neurosci. 2014 Feb 19;8:47. doi: 10.3389/fnbeh.2014.00047 (PMC3928569; doi:10.3389/fnbeh.2014.00047)

**Figure S3. Dendrogram resulting from a hierarchical cluster analysis in socially-housed rhesus monkeys.**

Following the multiple component analysis, the coordinates of the 35 socially-housed rhesus monkeys were submitted to a hierarchical cluster analysis. The level indexes (above 0.10) are indicated on the dendogram. One partition (blue arrow) resulted in 8 clusters for which the intra-cluster inertias are indicated on the right side of the graph. Animals from clusters 1, 4 and 7 (red font) expressed behavioural features similar to a few depressive symptoms whereas animals from the other clusters (green font) did not.


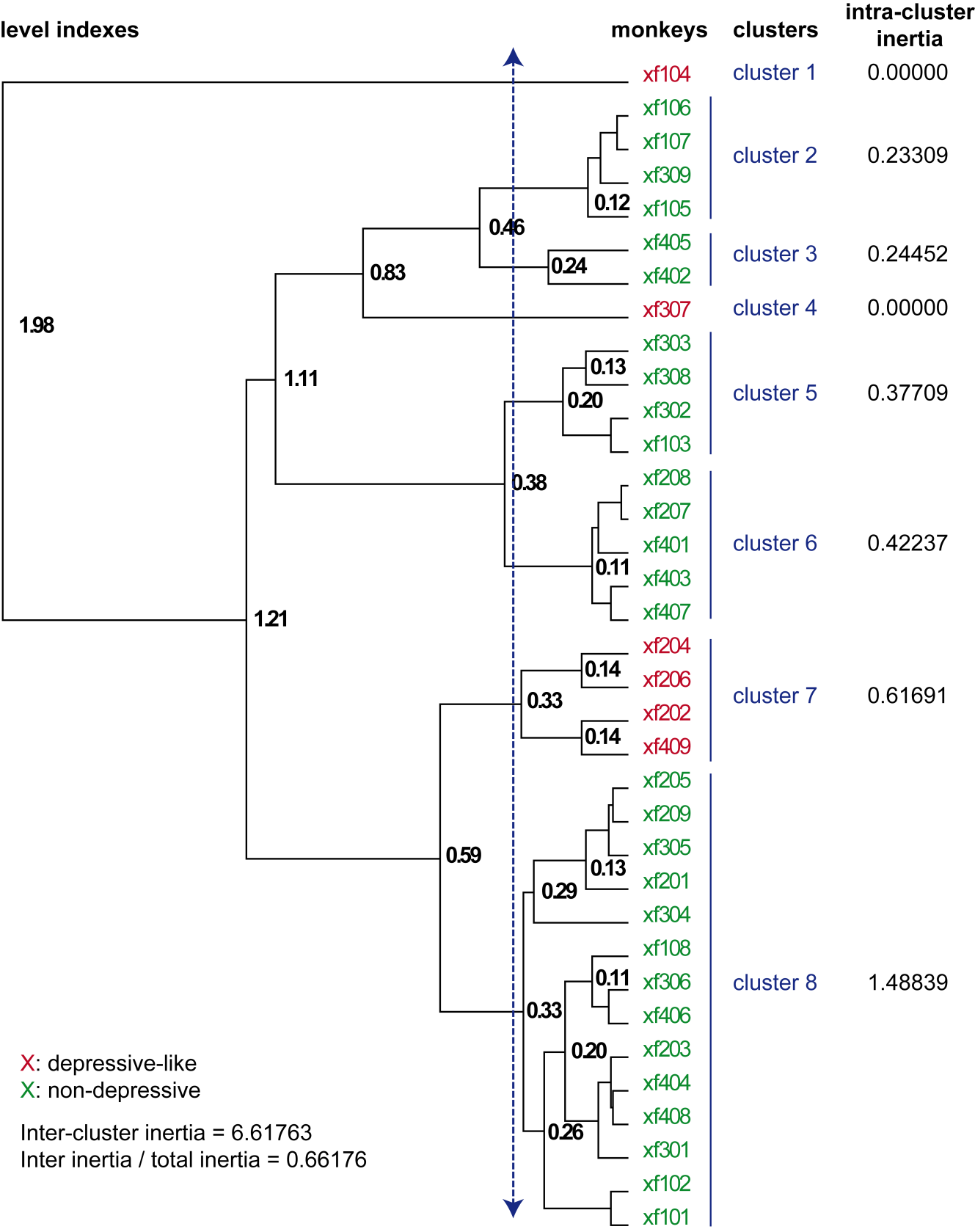

Supplement: Figure S3 — Dendrogram resulting from a hierarchical cluster analysis in socially-housed rhesus monkeys. Following the multiple component analysis, the coordinates of the 35 socially-housed rhesus monkeys were submitted to a hierarchical cluster analysis. The level indexes (above 0.10) are indicated on the dendrogram. One partition (blue arrow) resulted in 8 clusters for which the intra-cluster inertias are indicated on the right side of the graph. Animals from clusters 1, 4, and 7 (red font) expressed behavioral features similar to a few depressive symptoms whereas animals from the other clusters (green font) did not. [file DataSheet7.DOCX]
